# Activity patterns throughout the annual cycle in a long-distance migratory songbird, the red-backed shrike *Lanius collurio*

**DOI:** 10.1186/s40462-022-00355-0

**Published:** 2022-12-01

**Authors:** Pablo Macías-Torres, Thomas Alerstam, Arne Andersson, Johan Bäckman, Kasper Thorup, Anders P. Tøttrup, Sissel Sjöberg

**Affiliations:** 1 Department of Biology, Lund University, Ecology Building, Lund, Sweden; 2 Center for Macroecology, Evolution and Climate, Globe Institute, University of Copenhagen, Copenhagen, Denmark; 3 Natural History Museum of Denmark, University of Copenhagen, Copenhagen, Denmark

**Keywords:** Activity data, Accelerometer, Energy budget, Annual cycle, Biologging, Migration

## Abstract

**Background:**

Long-distance migratory birds undergo complex annual cycles during which they must adjust their behaviour according to the needs and conditions encountered throughout the year. Yet, variation in activity throughout the entire annual cycle has rarely been studied in wild migratory birds.

**Methods:**

We used multisensor data loggers to evaluate the patterns of activity throughout the complete annual cycle of a long-distance migratory bird, the red-backed shrike *Lanius collurio*. Accelerometer data was used to identify life-history stages and to estimate levels of activity during various phases of the annual cycle. In this study, we analysed the variation in daytime activity along the annual cycle and between migratory and non-migratory days.

**Results:**

The birds’ daytime activity varied throughout the annual cycle while night-time activity was almost exclusively restricted to migratory flights. The highest daytime activity levels were observed during the breeding season, while it remained low during autumn migration and the winter period. Daytime activity differed between sexes during the breeding period, when the males showed the highest level in activity. During migratory periods, both sexes exhibited a higher daytime activity in spring compared to autumn migration, being particularly high in the final migratory leg towards the breeding ground. The birds showed a lower daytime activity on migratory days (days when a migratory flight took place during the succeeding night) than on non-migratory days during both migratory seasons.

**Conclusions:**

Activity measured during daytime results from a combination of several behaviours, and a high daytime activity during spring migration and the breeding period is possibly reflecting particularly energy-demanding periods in the annual cycle of migratory birds. The use of multisensor data loggers to track annual activity provides us with a full annual perspective on variation in activity in long-distance migratory species, an essential approach for understanding possible critical life-history stages and migration ecology.

**Supplementary Information:**

The online version contains supplementary material available at 10.1186/s40462-022-00355-0.

## Background

The annual cycle of many organisms consists of a succession of life-history events separated by time and potentially location and underpinned by ecological and physiological processes. The main life-history events in birds are breeding, moult, and in migratory species, migration [37]. These energy-demanding events are scheduled within the annual cycle with as little overlap as possible between them [38]. Because the specific needs and conditions at each stage of the annual cycle might differ (e.g., fattening up prior to a migratory trip or finding extra food for raising the offspring), it is expected that animals adapt their behaviour—and the associated energy expenditure—accordingly. Furthermore, these events are inextricably linked as the outcome of one event might influence future life-history stages and ultimately fitness ([27]; but see: Brlík et al. [13]). Therefore, the study of annual cycles is highly relevant for the understanding of how a species manages its behaviour (and therefore their energy reserves) throughout the year so that they maximize lifetime reproductive success. Yet, there are few studies that take a full annual cycle perspective when studying animal ecology in bird species [35].

Migratory birds move between seasonal environments to find suitable conditions for breeding and non-breeding periods [5] while exploiting temporal and ephemeral peaks of resources on the route [4, 51]. It is important for migratory birds to adjust their behaviour according to environmental conditions on route, as well as in the wintering areas [11, 12, 52–54], as both internal (e.g. body condition; [34]) and external factors (e.g. resource availability [22, 23, 43, 44]) influence activity levels at specific times of the year.

Long-distance migration is regarded as a period with high energy demands, and probably also a period with high mortality [29, 46]. Hence, migrants must carefully allocate their resources to successfully complete all life-history stages. In the context of the annual cycle, the breeding period is suggested to be the most nutritionally demanding life-history event [17, 18, 20], where a large proportion of the annual energy budget is expected to be expended. In contrast, Piersma and Drent [40] estimated annual energy budgets for two populations of bar-tailed godwits *Limosa lapponica*, and concluded that daily energy expenditure for the migratory period exceeded that from the breeding period. In addition, energy budgets during migration depended on the migration distance of the two populations. Similarly, based on theoretical predictions, Alerstam [2] estimated that the annual energy expenditure for migration in arctic terns *Sterna paradisaea* exceeded that for breeding and feeding the young.

Understanding how wild animals allocate their resources throughout the annual cycle is of great interest for ecologists, as it affects the individuals’ survival and fitness, although this has been difficult to study in the wild due to the lack of technology. Over the last decades, the miniaturisation of logger devices has provided unprecedented data for quantifying movements of wild-living animals [14]. Recently, new insights into the levels of activity during the annual cycle of migratory species have increased with the use of accelerometer data [6, 7]. High-frequency acceleration data can be used to directly estimate energy expenditure in wild birds [14, 25, 56] and have for example been applied to assess differences in energetics and migration strategies between populations of the same species [23]. However, very few studies have measured activity patterns across different stages of the annual cycle in wild migratory birds [20]. Miniaturized accelerometer loggers offer a great opportunity to compare activity levels across the annual cycle of small-sized long-distance migratory songbirds.

Here, we study red-backed shrikes equipped with multisensor data loggers during the entire annual cycle. Previously, a first case study for this species using the same multisensor data loggers, and a review article including data from an additional individual, have been presented [6, 7], while in this study we focussed on studying the activity values recorded from several individuals. Recorded activity data allowed us to estimate levels of activity at different life-history stages throughout the year. Based on the activity data, we describe the annual activity pattern of the red-backed shrikes, distinguishing between day and night-time activity. Night-time activity was almost exclusively associated with migratory flights (continuous flapping flight) and could not be compared quantitatively with daytime activity (but allowed us to define different periods in the migratory seasons; see methods). Hence, in this study we focus on the variation in daytime activity, which includes the foraging activity needed to cover the full energy demands of the birds in addition to energy-demanding activities for breeding, migration etc., and compared activity levels during different events of the annual cycle as well as between sexes. Furthermore, we investigated if daytime activity levels differed between autumn and spring migration, and between migratory (i.e., days when a migratory flight took place during the succeeding night) and non-migratory days. We also analysed daytime activity patterns in the period immediately after arrival at breeding grounds.

Given the lack of comprehensive information about variation of activity during the annual cycle of migratory birds, an important objective of this study was to start exploring the overall annual pattern of activity and try to interpret peaks and valleys in activity level at different periods of the year. In addition, we aimed to test two specific and major hypotheses: (1) It has been claimed that for nidicolous/altricial birds, where parents feed their nestlings and fledglings up to their independence well after fledging, the breeding season represents the period of the year when the adult birds reach their maximum activity and exertion levels. During this period the birds are under very strong selection to maximize their reproductive success, demanding very high activity levels to obtain mates and territories, produce eggs and feed the offspring [8, 17, 18, 20]. We therefore test if the breeding period in a long-distance migratory species comprises a period of high exertion compared to other periods of the annual cycle. In addition, (2) we also tested the hypothesis that migratory birds are more time constrained and thus migrate faster during spring compared to autumn migration, because of the strong competition for breeding resources [3, 30, 39]. This means that during spring migration birds are strongly favoured by early arrival at breeding sites before their competitors and they are thus expected to adopt time-selected migration strategies involving e.g., sprint migration. Fast migration is expected to be accomplished by high fuel deposition rates at stopover sites which in turn will be obtained through intensive foraging and thus high diurnal activity levels are expected [33]. Hence, it is predicted that daytime foraging activity is more intensive during spring than autumn migration, particularly in the final part of spring migration when the birds may adopt a sprint migration strategy [3, 30].

## Methods

Data from 14 red-backed shrikes were included in this study (11 males and 3 females). Birds were captured in their breeding territories using mist nets or spring traps between May–July of 2014–2020 in Gribskov, Denmark (55.98°N, 12.33°E). Captured birds were ringed with aluminium rings and colour-rings with individual alphanumeric inscription. Small multisensor data loggers (< 1.2 g, representing 3.6–4.7% of body mass when deployed; [6]) were fitted as backpacks with a leg-loop harness. These multisensor data loggers incorporate a light sensor, an accelerometer, a temperature sensor, and a barometric pressure sensor. However, in this study only data from the accelerometer is included. The multisensor data loggers were designed so they record data every day for at least one calendar year [6]. Birds were recaptured 1–2 years after the loggers were deployed and the stored information was retrieved from the data loggers.

### Accelerometer data

The multisensor data loggers included an accelerometer that measured acceleration on a single axis (approximately vertical when the bird is flying) and were customized to identify activity. A total of 12 samples were recorded per hour (one sample every 5 min). At each sampling event, 5 subsamples (100 ms duration at 100 Hz) with 5 s spacing time were recorded. If all five subsamples detected no movement (acceleration varied less than 1/4 g), the sample scores a "0", and if all subsamples showed active movement, it scores a "5". Intermediate cases gave scores from "1" to "4". The exact sampling method involves several steps, and a detailed explanation of the data logger’s operation is presented in Bäckman et al. [6]. Red-backed shrikes are described as sit-and-wait hunters, perching for long times and looking out for prey (mainly insects) to be captured mostly on the ground [57]. Therefore, daytime activity detected by the accelerometer in the red-backed shrikes should be considered as a combination of activities such as resting, preening, hunting, intra- or interspecific interactions, and short flights between perching sites.

Data for the full annual cycle (from the breeding season when the logger was deployed until the next breeding attempt when the logger was retrieved) was available for 12 out of 14 individuals. Data loggers from 2 individuals stopped working in the beginning of spring migration and only data until the last day of the winter period was used in this study. Individual “*148*”, ringed in 2014, had several 5-day periods with missing activity data due to a technical problem when light- and activity measurements overlaped. All samples during these periods were deleted. Another individual, “*F24*”, showed a few missing 5-min samples throughout the year (a total of 33 samples of 5 min), but the effect was minimal compared to the total number of samples and no data was deleted or modified for this individual (i.e., 33 samples with missing data were treated as samples of zero activity to keep a constant 24-h sample size). A total of four individuals were recaptured two years after the logger was deployed. For these birds, data was available for two years, but the quality of the data decreased in the second year due to battery issues. Because of the decrease in data quality, and to avoid pseudo-replication, only the first year of data was included in this study.

### Identification of the annual cycle stages

Every day was categorised into 12 periods based on each individual actogram (Table 1, Additional file 1: Figure S1), separating between migratory and non-migratory days. These periods were defined to distinguish between stages in the annual cycle and approximate geographical locations of the red-backed shrikes [52, 53]. Days in which red-backed shrikes performed, or did not perform, a migratory flight during the succeeding night, were categorized as migratory and non-migratory days respectively. We grouped the migratory days into five major migratory periods, three during autumn migration and two during spring migration. Different migratory periods were separated by major stopover periods (see below: “stopover”). Two migratory days were included under the same migratory period if there was less than 9 non-migratory days between them (see below: “stop-and-go”, Additional file 1: Figure S1). This 9-day threshold is based on a visual inspection of the individual actograms for grouping migratory days in different main migratory periods (Table 1). Spring migration was an exception as some individuals had very short stopover periods (only 4-day stopover in some cases), and the separation between the two migratory periods during spring was made by visual inspection of the actograms looking for the longest possible stopover period by the end of April. Given the big variation in the definition of a stopover [55], we categorised non-migratory days either as “stopovers” (long temporal duration gaps between migratory periods) or as “stop-and-go” days (days included in a migratory period during which the bird did not fly at night; Table 1). Furthermore, some individuals performed solitary nocturnal flights in the middle of a stopover period (Additional file 1: Figure S1; See “odd flights” in [7]) and those days were excluded from any category since we are unsure of the motivation behind them. All 12 periods were assigned specific coordinates based on the already known migratory route of the same population of red-backed shrikes, which has been observed to show very little variation in route and timing of migration (see table 1 in [6, 7, 52, 53]). The coordinates for a migratory period were estimated as the mid-point between two stopovers. These 12 periods were grouped into the 4 main events in the annual cycle: autumn migration, spring migration, summer and wintering (Table 1).

**Table 1:** Categorisation of annual cycle events and periods in red-backed shrikes based on their individual actograms

Annual cycle event	Period name	Description	Coordinates
Autumn migration	MA1	First migratory period during autumn migration. From Denmark to South Europe	N: 50°; E: 16.15°
	MA2	Second migratory period during autumn migration. Sahara crossing	N: 27.5°; E: 25°
	MA3	Third migratory period during autumn migration. From Sahel to South Africa	N: − 7°; E: 25°
	Stopover 1	First stopover during fall migration in South Europe	N: 44°; E: 20°
	Stopover 2	Second stopover during fall migration in Sahel region	N: 11°; E: 30°
	Stop and go autumn	Non-migratory days inside a migratory bout. Not considered stopovers because of their short duration	Same coordinates as the migratory period it belongs
Wintering	Winter grounds	Stationary period between MA3 and MS1. South Africa	N: − 25°; E: 20°
Spring migration	MS1	First migratory period during spring migration. From the first nocturnal flight from the winter quarters until the longest stopover (stopover3) that take place in late April-beginning of May	N: − 17°; E: 31.5°
	MS2	Second migratory period during spring migration. From stopover 3 until the arrival at the breeding grounds in Denmark. Split into two segments to minimise the error estimates in sunrise and sunset times based on coordinates	First segment N: 22.5°; E: 43°; Second segment: N: 46.5°; E: 22.75°
	Stop and go spring	Non-migratory days inside a migratory bout. Not considered stopovers because of their short duration	Same coordinates as the migratory period to which it belongs
	Stopover 3	Third stopover period during spring migration in Northeast Africa	N: 8°; E: 43°
Summer (breeding)^a^	Summer	Days at the breeding site in Denmark	N: 56°; E: 12.3°
–	Odd flights	Isolated flights performed in the middle of a stopover	Same coordinates as the migratory period to which it belongs

^a^Days included under the summer period includes two parts of the breeding seasons: both after a logger was deployed (first summer) and before it was recaptured a year later (second summer)

### Daytime and night-time activity

To account for differences in day length between different periods, sunrise and sunset times were calculated for each day to distinguish between daytime and night-time activity, based on the estimated coordinates for that period. Daytime activity consisted of the sum of the hourly accelerometer values between sunrise and sunset (rounded to the nearest integer hour) for a given date and coordinate. In very few cases, migratory flights were prolonged after sunrise, but only activity after landing (i.e., when the accelerometer stopped recording the highest possible levels of activity) is considered as part of daytime activity for that specific day.

Night-time activity was mainly absent throughout the year, except for migratory days. When red-backed shrikes performed migratory flights, the accelerometer recorded maximum activity values continuously for several samples (i.e., score 4–5 per sample along several consecutive 5-min samples). To summarise migratory flights, we calculated the migration duration per night by summing up all the 5-min periods indicating continuous flight, similar to what has been described in Bäckman et al. [7].

In this study we have focused on comparing patterns in daytime activity at different stages of the annual cycle. While daytime activity is present all year round, night-time activity was almost only present during migratory days (as explained above). In addition, daytime and night-time activity could not be directly compared as the former involves both energy input and output while the later only relates to energy expenditure during continuous flapping flight. Also, due to the data logger sampling limitations, there is no linear scale between the activity values recorded for nocturnal and diurnal activity, with activity values reaching the cut-off maximum levels for all intervals of continuous flapping flight (night-time activity). Then night-time activity could not be compared quantitatively with daytime activity (but allowed us to define different periods in the migratory seasons). Thus, we investigated the quantitative patterns in daytime activity throughout the annual cycle and treated night-time activity as a binomial variable as described above: migratory flight or non-migratory flight days (i.e., how daytime activity is influenced by having migratory flights on preceding nights).

For a detailed evaluation of the daytime activity upon arrival at the breeding grounds we considered a period of 10 days after the arrival date. This time period is based on the average days that females have been observed to spend between arrival and egg laying: between 8.6 and 12.6 days depending on the habitat [49], and about 8 to 15 days when looking at the female actograms included in this study (visually exploring a reduction in the activity recorded at the breeding grounds). However, we have considered a shorter period of 4 days after the arrival date when comparing the activity between early and late arriving individuals since we expect that the main competition for territories and mates happens some days before the first egg is laid.

### Statistical analysis

Mean daytime activity and standard deviation were calculated for all 12 periods. We conducted ANOVA analyses based on linear mixed effect models to test for differences in daytime activity between events in the annual cycle, including daylength and sex as fixed effects and individual as a random effect. The variable “year” was initially included in the models but removed in the end as the models performed better without it (they had a lower AIC). Daytime activity values were square root transformed to follow model assumptions of normally distributed residuals. Because the activity values recorded by the accelerometer were repeatedly sampled through time, we also accounted for temporal autocorrelation, as the activity of one day might be influenced by previous observations, and followed the guidelines of Mitchell et al. [36]. Hence, we fitted a first-order autocorrelation, which computes a correlation coefficient between the residual of any given day and the residual of the preceding day. Because the total amount of hours in daytime (i.e. day length) might influence the values of activity [43], we included day length as part of the models explaining daily activity. Yet, in all analyses we used total values of daytime activity (i.e., the sum of activity from sunrise to sunset); changing activity values to activity per hours of day length (dividing the total daytime activity by the numbers of daylight hours at different times of the year) did not change the annual pattern in diurnal activity (Additional file 1: Figure S3).

All statistical analyses were performed in R.3.6.2 [50]. R-packages “nlme” [42] was used to perform linear mixed effect modelling including an autocorrelation factor, while “emmeans” [32] was used to calculate first estimated marginal means and to conduct pairwise post-hoc comparisons of daytime activity between events in the annual cycle. Sunrise and sunset were calculated using function *sunriset* in the R-package *maptools* [9].

## Results

### Overview of the annual activity patterns

**Fig. 1:**
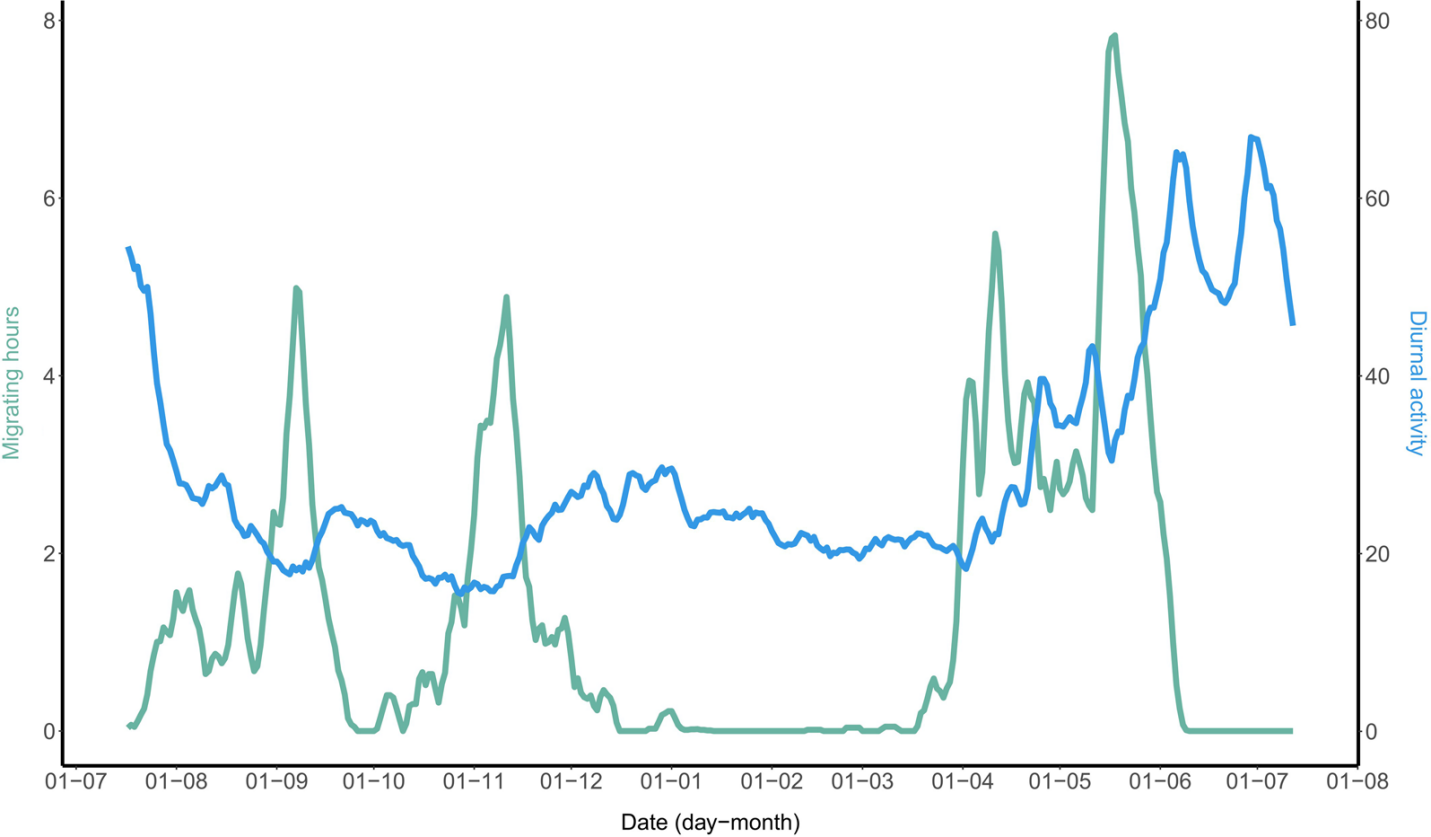
Average daytime activity and number of hours on migration ofred-backed shrikes throughout the annual cycle, based on accelerometer data for 14 individuals. While daytime activity varied throughout the entire year, nocturnal activity (continuous flapping flight) occurred only when migrating. Solid lines illustrate a 5-day running mean for all individuals per variable (green: migrating hours; blue: daytime activity). Data points and confidence intervals for the daytime activity are shown in Fig. 2b

**Fig. 2:**
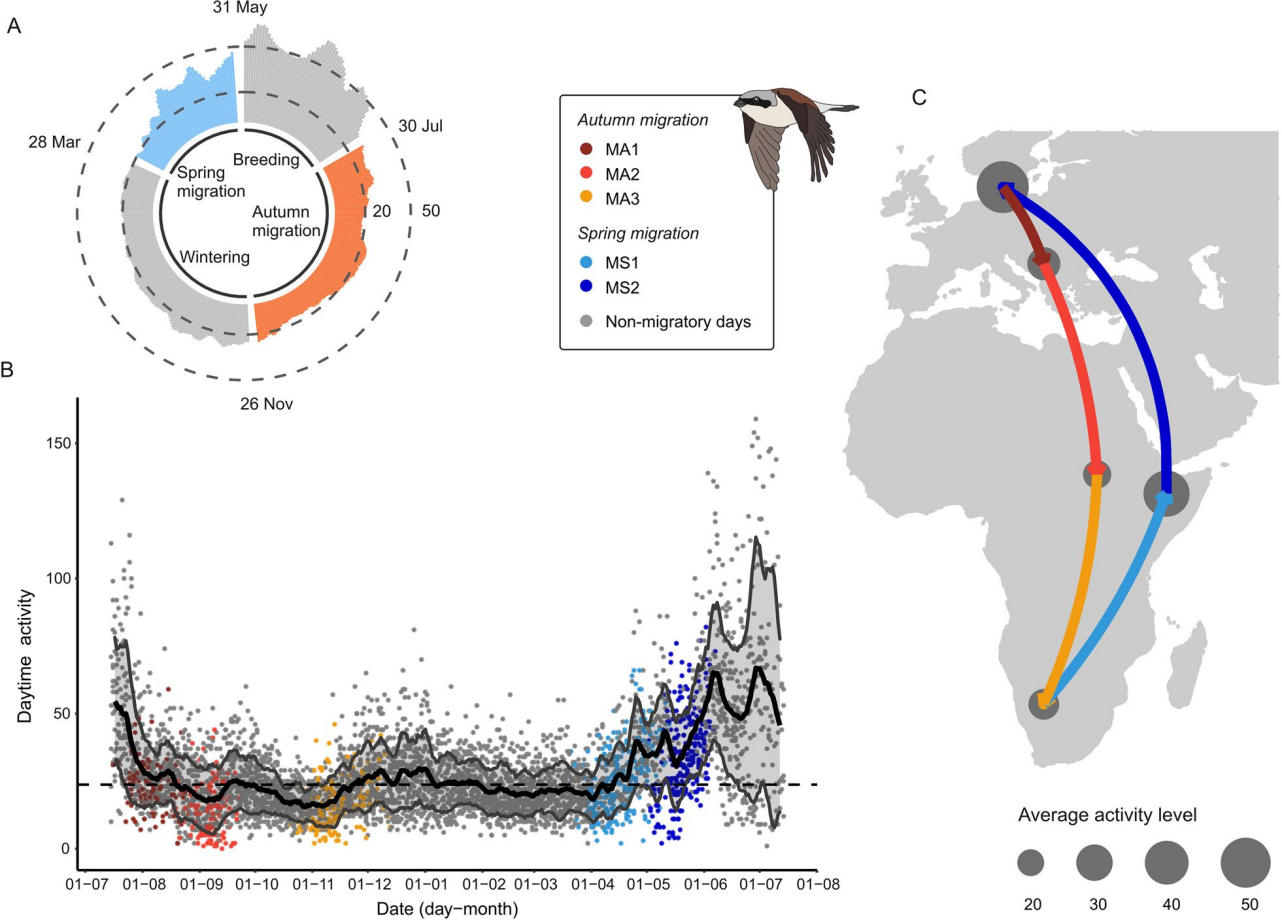
Daytime activity at different locations and phases of the annual cycle in red-backed shrikes. **A** Mean daytime activity throughout the annual cycle with migratory periods shown in colours (spring migration in blue and autumn migration in orange). Dates indicate the mean starting and finishing dates for each migratory period for all the individuals. Two dashed lines serve as guiding for daytime activity values of 20 and 50. **B** Same mean daytime activity values with a 95% confidence interval shown (shadowed area). Each point represents the activity value per day per individual. Autumn and spring migration are divided in three and two migratory legs respectively, shown in different colours. Dashed line shows the mean value of activity during the winter period as a reference. **C** The map represents the known migratory route of the same population of red-backed shrikes [52, 53]. Coloured arrows indicate migratory periods with the same colours as **B**. Mean activity during a stopover period is illustrated by the size of the grey circles at each stopover location

**Fig. 3:**
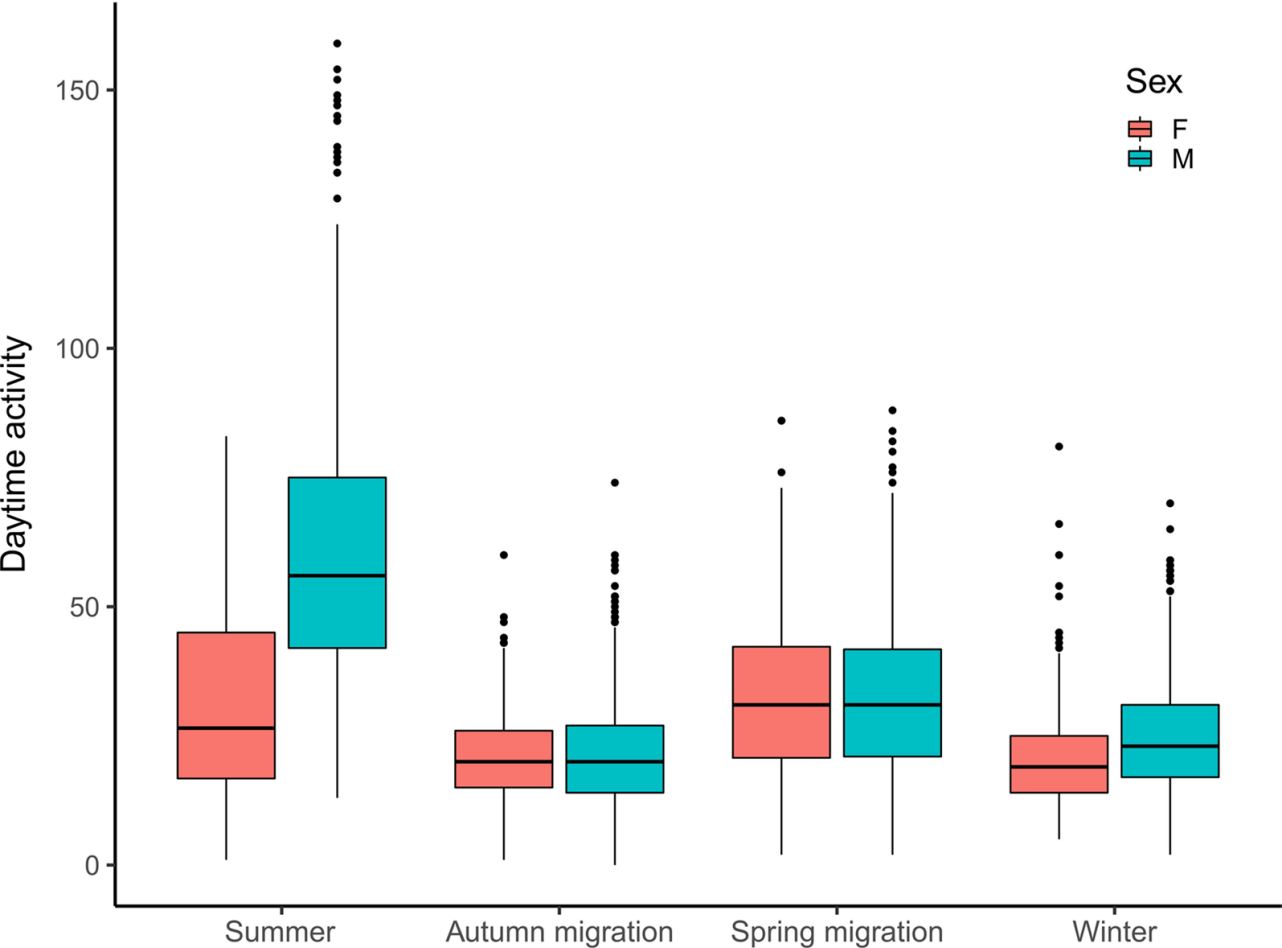
Daytime activity of red-backed shrikes at different events of the annual cycle. Between sexes, daytime activity was different only for the summer (breeding period), but statistically identical for the others. Pairwise post hoc comparison for male red-backed shrikes (n = 11) showed that all four events of the annual cycle were statistically different from each other, while for females (n = 3) only spring migration was statistically significant from all other events (Additional file 1: Table S2)

Daytime and night-time (i.e., migratory flights) activity varied throughout the annual cycle in the red-backed shrikes (Fig. 1; Additional file 1: Table S1). The birds were almost exclusively active during daytime except when undertaking migratory flights, when maximum activity levels were recorded by the data logger (Fig. 1; Additional file 1: Figure S1). Daytime activity varied depending on the time of the year and the location of the birds (Fig. 2) and differed significantly between the 4 main events of the annual cycle (Fig. 3; sqrt(Activity) ~ events * Sex + daylength, random =  ~ 1|Individual, correlation = corAR1(value = 0, form =  ~ day|Individual); ANOVA, events: F_3,4686_ = 240.58, *p* < 0.0001). We also observed a significant interaction between events of the annual cycle and sex (Table 2; ANOVA, events:Sex: F_3,4686_ = 31.93, *p* < 0.0001): males significantly differed in activity among all four events (*p* < 0.001; Fig. 3; Additional file 1: Table S2); while females only showed statistically significant differences between spring migration and autumn migration, spring migration and summer and spring migration and winter (*p* < 0.001; Fig. 3; Additional file 1: Table S2). When only daytime activity values for both sexes together were considered, the red-backed shrikes showed the highest daytime activity during the summer period followed by spring migration, winter and autumn migration (Additional file 1: Table S1; Mean daytime activity ± SD for breeding: 53.8 ± 29.1; spring migration: 32.3 ± 15.6; winter: 23.7 ± 10.3 and autumn migration: 21.1 ± 10.0).

**Table 2:** Summary of the statistical model showing the relationship between daytime activity and the four main events of the annual cycle (summer, winter, autumn migration and spring migration)

Predictors	Estimates	95% CI	DF	*p* value
(Intercept) [ref. Autumn migration]	− 0.07	− 0.83 to 0.68	4686	0.847
**Events [Spring migration]**	**0.84**	**0.49** to **1.19**	**4686**	** < 0.001**
**Events [Summer]**	− **0.48**	− **0.89** to − **0.08**	**4686**	**0.020**
Events [Winter]	− 0.06	− 0.34 to 0.23	4686	0.706
Sex [M]	0.27	− 0.35 to 0.90	12	0.357
**Daylength**	**0.33**	**0.29** to **0.37**	**4686**	** < 0.001**
Events [Spring migration] * Sex [M]	− 0.12	− 0.53 to 0.28	4686	0.543
Events [Summer] * Sex[M]	**1.94**	**1.52** to **2.36**	**4686**	** < 0.001**
Events [Winter] * Sex [M]	0.29	− 0.03 to 0.62	4686	0.078
**Random effects**				
Std dev	0.4	0.26 to 0.62		
N Individual	14			
Observations	4707			
Marginal R2/Conditional R2	0.382/0.451			
Correlation Structure: ARMA(1,0) Formula: ~ day | Individual				
Phi	0.54	0.52 to 0.56		

Statistically significant parameters are highlighted in bold. Model formula: sqrt(Activity) ~ events * Sex + daylength, random =  ~ 1|Individual, correlation = corAR1(value = 0, form =  ~ day|Individual)

### Migration

**Fig. 4:**
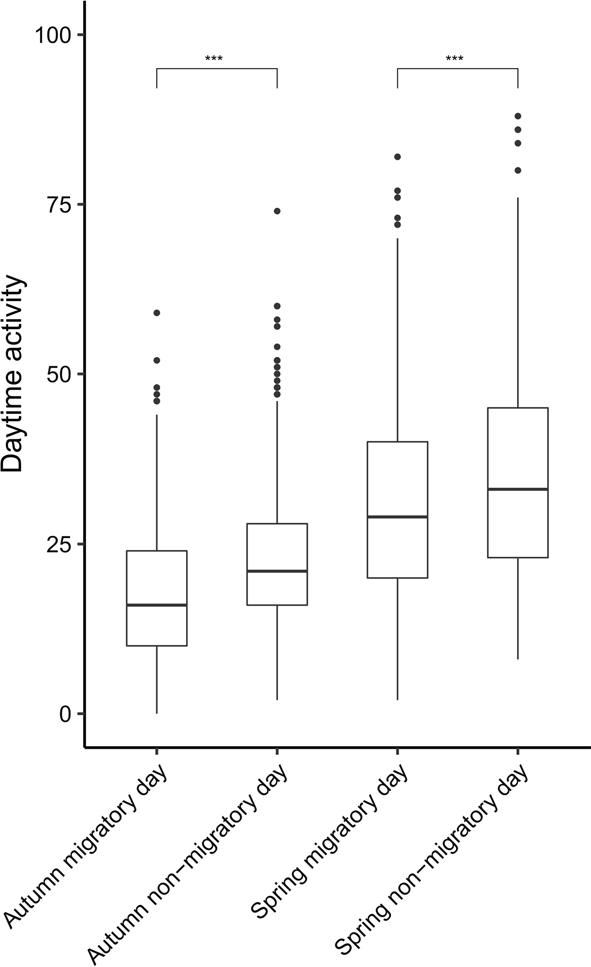
Differences in daytime activity between migratory and non-migratory days in autumn and spring migration. Red-backed shrikes were less active during migratory days compared to non-migratory days. Non-migratory days includes stopovers and “stop and go” days; while migratory days are those in which the red-backed shrikes migrated during the evening. Post-hoc Tukey HSD results are shown with stars over the boxplots showing significant differences at a level of *p* < 0.001

Daytime activity was lower during migratory days compared to non-migratory days (i.e., both stopover and stop-and-go) during both autumn and spring migration (Fig. 4; sqrt(Activity) ~ Migration + daylength + 1|Individual, Correlation Structure: ARMA(1,0). ANOVA, Migration: F_3,2384_ = 76.79, *p* < 0.0001. Post-hoc Tukey HSD test showed significant differences in mean activity between the 4 groups at 0.001 level of significance).

Daytime activity differed between different legs of migration, with the activity during the second leg of autumn migration (i.e., the crossing of the Mediterranean Sea and the Sahara Desert) being the lowest (daytime activity mean ± SD for MA1: 23.3 ± 9.85; MA2: 14.0 ± 9.73; MA3: 16.6 ± 8.68; MS1: 26.6 ± 12.7 and MS2: 35.5 ± 16.3; Additional file 1: Table S1)*.* In autumn migration, red-backed shrikes kept a constant activity throughout the first two autumn migratory periods, while it increased along the last autumn migration leg until the arrival at the winter grounds (Fig. 2; Additional file 1: Figure S2; Activity ~ date + daylength + 1|Individual; model estimate (95% CI) for date in MA1: 0.03 (−0.29–0.35), *p* = 0.86; MA2: 0.14 (−0.06–0.33), *p* = 0.25; MA3: 0.25 (0.15–0.34), *p* < 0.001). Overall, daytime activity increased daily during spring migration; a linear increase was observed during the first segment of spring migration (MS1) and remained high throughout the second leg upon arrival at the breeding grounds (Activity ~ date + daylength + 1|Individual; model estimates (95% CI) for date in MS1: 0.33 (0.20–0.46), *p* < 0.001; MS2: 0.31 (−0.01–0.62), *p* = 0.11; entire spring migration: 0.09 (0.02–0.15), *p* = 0.025; Fig. 2; Additional file 1: Figure S2). These high values in daytime activity during the second leg of spring migration took place while the birds migrated for several consecutive nights until the arrival at the breeding grounds (some individuals migrated up to 22 nights in a row: see MS2 in Additional file 1: Figure S1).

### Breeding period

**Fig. 5:**
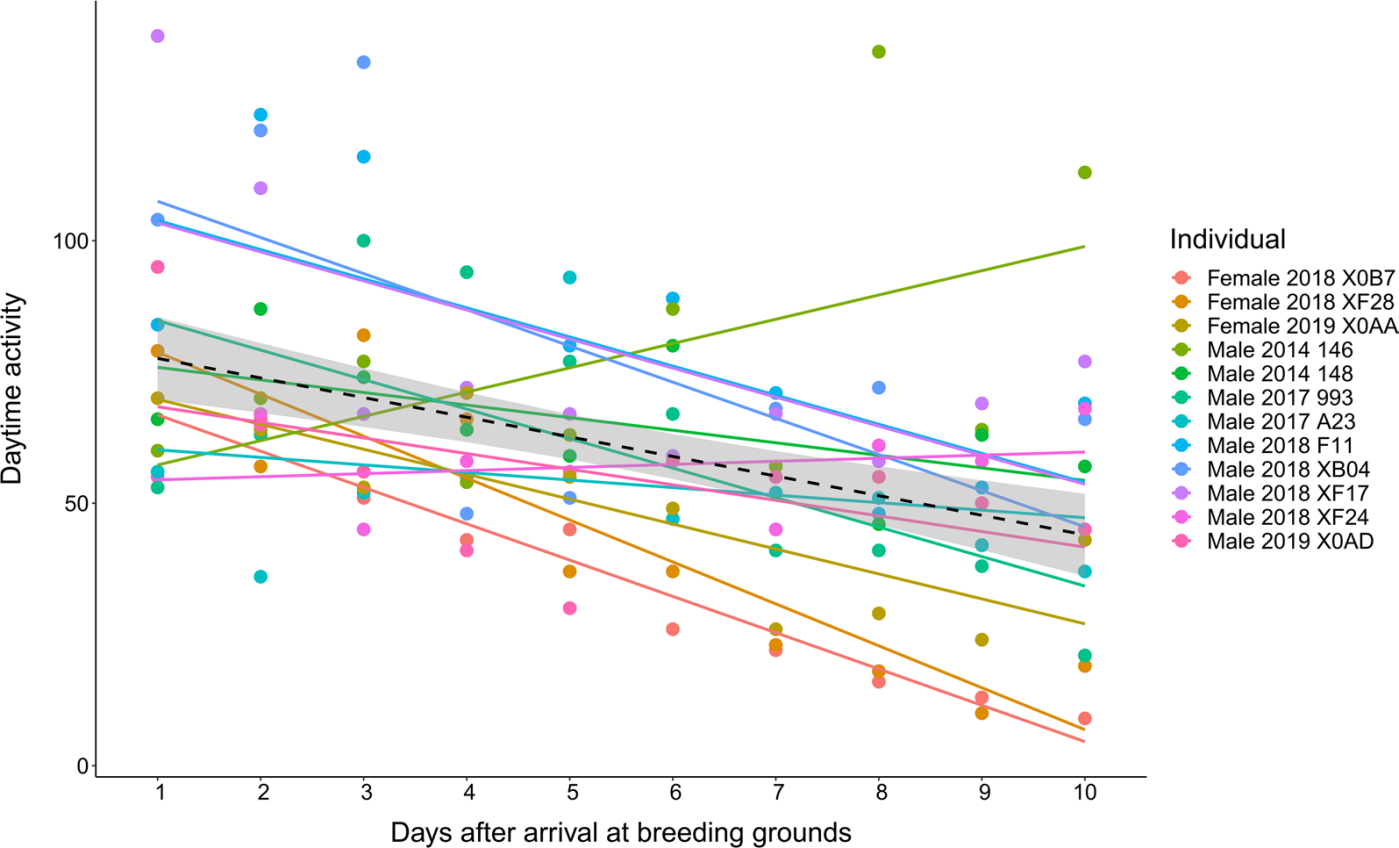
Daily daytime activity in red-backed shrikes over a 10-day period after the arrival at the breeding grounds. Red-backed shrikes showed high activity upon arrival at the breeding grounds, gradually decreasing during a 10-day period after that. Dashed line represents the regression line for all individuals. Individual regression lines are indicated in colours

Following spring migration, the red-backed shrikes arrived at their breeding territories and showed some of the highest scores in activity (Fig. 2). Daytime activity statistically differed between males and females during the breeding period (Fig. 3; Table 2). After arrival at the breeding grounds the birds’ daytime activity decreased along a 10-day period (Activity ~ date + 1|Individual; F_1,104.3_ = 30.75, *p* < 0.001; Fig. 5). However, we found no evidence for a difference in the activity during a 4-day period upon arrival between early and late arriving individuals (4-day mean activity ~ date; linear model with only males included: *F*_1,7_ = 3.54, p = 0.10; linear model both males and females: *F*_1,10_ = 1.24, *p* = 0.29; Arrival range: May 24 to June 7). Also, the highest daytime activity values were recorded during the mid-breeding period and referred to male red-backed shrikes (beginning of July, Fig. 2b).

## Discussion

Our data from miniaturised data loggers revealed large differences in daytime activity throughout the year in a long-distance migratory songbird, the red-backed shrike. The varying patterns in daytime activity are important for understanding the costs and constraints associated with different annual cycle events [10, 35], and underlines the complexity of the annual cycle in a long-distance migratory songbird. However, it is difficult to accurately assess exactly what the patterns of low or high values in daytime activity means. Possibly, an increase in activity relates to an increase in the energy demands and/or lower food availability in the surroundings [22, 44]. Yet, it should be noted that in this study we have only measured differences in activity and not specific behaviours.

### Breeding

The most energetically demanding days in the annual cycle of birds are thought to be found during the breeding period, mainly due to the chick rearing phase [8, 18, 31]. In addition, observational studies of birds in the wild have inferred a higher daily energy expenditure during the breeding season compared to non-breeding season [17, 20]. Correspondingly, the red-backed shrikes in this study changed their daytime activity between different phases of the annual cycle, being most active during the breeding period. To successfully raise offspring, the red-backed-shrikes have to secure and defend a territory, find a mate, build a nest, feed the chicks and attend the fledglings. Together these behaviours resulted in higher levels of activity than those displayed in any other stage (except migratory flights). Although we made no direct field observation to compare the values of the accelerometer, we believe that the peak of activity observed in the mid-breeding period has to do with active feeding of the hatchlings. Drent and Daan [18] argue that the maximum sustained working level done by breeding parents when trying to maximise their fitness is approximately four times the basal metabolic rate. The red-backed shrikes in this study more than doubled their daytime activity during the time spent on the breeding grounds compared to the activity shown at the wintering grounds, indicating a dramatic increase in workload (assuming a positive relationship between activity metrics and energy expenditure) during the breeding period. This is in accordance with previous studies that found an increase in the activity of red-backed shrikes while feeding the young compared to the activity shown at the non-breeding areas [16].

On the other hand, migration-related activities have been discussed to exceed the energy budget of breeding in a long-distance migratory wader species [19] and the arctic tern [2]. However, we observed that the levels of daytime activity were overall higher in the breeding period than during migration, indicating a higher energy demand during this period compared to migration, which is in accordance with other studies estimating energy expenditure in bird in the wild [20]. It should be noted that the godwits studied in Drent and Piersma [19] is a nidifugous species and there is no active feeding by the parents, while parent red-backed shrikes actively feed their chicks both during nestling and fledgling periods [57], likely resulting in different energy expenditure during the breeding period.

Although the daytime activity remained almost identical between both sexes throughout the non-breeding periods, females had a lower activity compared to the males during breeding phase. The decreased activity in females is probably caused by females incubating almost exclusively [57]. This does not necessarily mean that females had a lower energy expenditure during the incubation phase, since maintaining the eggs at temperatures suitable for embryonic development is energetically costly [21].

### Wintering

Daytime activity during the wintering period was consistently low compared to other events of the annual cycle, with no remarkable changes in the activity patterns from arrival to the winter areas until the onset of spring migration. This consistency in low activity is possibly due to the low-energy demanding activities that take place during this time. Red-backed shrike’s behaviour during the wintering period has previously described as relaxed, spending around 80% of the day motionless [16]. Red-backed shrikes moult flight feathers during their stay at the winter grounds [15], which might constitute the most energy demanding activity during this period. Also, many species of shrikes (Lanidae family) actively defend their territories outside of the breeding area [16, 47], which might also constitute an energy demanding activity during this period. We argue that the activity observed during the wintering period is the result of mostly survival activities (i.e., avoiding predators and feeding until the onset of spring migration), when almost no extra energy-costly activities (besides feather moult) is performed. Thus, the mean activity level observed during this period could serve as a good reference value to assess if other periods in the annual cycle are particularly energy demanding.

### Migration

Daytime activity differed between the migratory periods (Figs. 1, 3), as daytime activity was significantly lower during the autumn compared to the spring migration period. This is probably related to the differences in the time spent on migration between those two seasons, with a faster (but longer) spring migration [52, 53]. During spring migration, the red-backed shrikes in this study gradually increased their daytime activity. This was concurrent with migratory flights occurring almost every night during the final part of spring migration (MS2 in Additional file 1: Figure S1) until arrival at the breeding grounds (i.e., from the Arabian Peninsula across Middle East and Europe). During migration, most of the time is expected to be spent at stopovers [28], yet, the red-backed shrikes seem to avoid stopping over for long periods as they approach the breeding grounds and, thus increasing the speed of the last segment of spring migration. This pattern is similar to the sprint migration strategy [3] also reported in other species [11, 12], where the birds, at least those in good condition, try to reach their breeding sites as soon as possible by increasing their workload and combining foraging and replenishment of fuel reserves during the day with migratory flight during the night. The accelerometer data in this study thus suggests that red-backed shrikes behave strikingly differently in spring and autumn migration, adopting different migration strategies, probably due to different selection pressures [39]. Another prediction associated with sprint migration is that the birds would be expected to fly at an increased airspeed [3, 39] but this could not be tested because the variation in speed/intensity/power of flight is not indicated by the accelerometer data, showing maximum cut-off activity levels for all intervals of continuous flapping flight. Still, we believe that daytime activity is indicative of the needs encountered during both diurnal as well as nocturnal periods. Thus, our finding of a higher daytime activity during the spring migration compared to autumn migration suggests that that the former is more time constrained and that the birds work harder during this period.

The red-backed shrikes in this study had a lower daytime activity during migratory days compared to non-migratory days, particularly during the autumn barrier crossing of the Sahara Desert, when the birds showed the lowest activity within the annual cycle. This suggests that red-backed shrikes spend more time resting during daytime when performing a migratory flight the succeeding night, although foraging events in the Sahara Desert might occur if suitable habitats are encountered [1]. The lower daytime activity shown during migratory days might reflect the compensation for lack of sleep from continuous nocturnal flights as taking naps during daytime is a common behaviour observed in nocturnal migrants [24].

### Arrival at the breeding grounds

Early arrival at the breeding grounds has been linked to a higher territory quality and reproductive performance [30, 48, 49]. Daytime activity upon arrival at the breeding grounds was among the highest observed, and it decreased in the following days. This peak in activity can be related to the intense competition for territories and mates upon arrival. The high daytime activity upon arrival at the breeding grounds is preceded by another peak of activity during the last leg of the spring migration, suggesting that the levels of exertion performed by the birds during these periods is among the highest found during the annual cycle, and might be crucial for the reproductive success and or survival of the birds. However, the fact that we did not observe any differences in activity between early and late individuals upon arrival at the breeding grounds might indicate that this arrival period is equally intense for all the red-backed shrikes independently of their order of arrival in the season.

Assuming that the high levels of daytime activity shown by the red-backed shrikes during the breeding season and later part of spring migration are of general occurrence among long-distance migratory nidicolous/altricial birds, like e.g., songbirds and raptors, one might expect that these periods also would be peak periods of mortality risks for the migrants (if a bird moves around more it might become more easily detectable by a predator). The few studies existing that analyse when migratory birds die during the annual cycle indicate that increased mortality risk levels prevail during migration seasons compared to the stationary breeding and wintering periods [29, 46]. Although the causes of mortality during migratory periods remain mostly unknown [29], the high levels of daytime activity found in this study might indicate that birds expose themselves to increased mortality risks in order to make it to the breeding ground on time. Correspondingly, a low level of mortality risk during the breeding season [29, 46] is surprising if the breeding period is associated with the highest intensity of activity throughout the year. Still, there is perhaps a possibility to reconcile a high activity level with a low mortality risk during the breeding period: a carry-over effect of increased mortality into the succeeding post breeding periods [11–13]. Parent birds may work themselves close to exhaustion due to their intensive breeding activities [18], postponing the acute mortality risks into the post breeding season (autumn migration) when the adult birds may face problems of recovery from their exhausted state at the same time as they must deposit fuel for the autumn migratory journey in competition with a new generation of juveniles at the stopover feeding grounds.

## Conclusions

We found three prominent peaks in daytime activity level (activity level exceeding 30; cf. Figs. 1, 2) during the annual cycle: (1) the final 3–4 weeks of spring migration up to the arrival at breeding grounds. The high daytime activity during this period coincided with top levels of nocturnal flight activity (Fig. 1), probably making final spring migration a very hectic period in the red-backed shrikes’ annual cycle. However, even higher levels of daytime activity prevailed throughout the breeding season with two peaks, (2) at and immediately after arrival at the breeding site (when much activity was presumably devoted to territory establishment and pair formation) and (3) about a month later, at midsummer, when the parent red-backed shrikes probably had a high workload feeding their young. The lowest daytime activity levels (< 20; Figs. 1, 2) occurred during two periods of the autumn migration, coinciding with (1) the shrikes’ passage of the Mediterranean Sea and Sahara Desert (first half of September) and (2) their migration from Sahel to the final winter destinations in southern Africa (from mid-October to mid-November). Presumably, the shrikes had stored extensive fuel reserves for these travel segments during preceding stopover periods, allowing them to spend the days between the nocturnal flights at reduced daytime activity levels.

We conclude that the annual activity pattern for the red-backed shrikes supports the two major predictions about (1) maximal activity/workload during daytime at the breeding grounds and (2) significantly elevated activity levels during spring compared to autumn migration. The difference in activity levels during spring and autumn migration found in this study support the hypothesis of a time-selected migration in spring because of the advantages associated with early arrival at breeding sites in the competition for breeding resources [3, 30, 39]. In accordance with these two major hypotheses the elevated activity during the breeding season and the final half of spring migration gives a prominent support for distinctly higher exertion levels during these two periods that appear to be busier and more stressful for the shrikes than any other period of the year.

The use of a multisensor data loggers with accelerometer in this study have provided insight into the patterns in activity in a long-distance migratory songbird throughout different life-history stages, identifying periods with high daytime activity, where selection pressures might be stronger (e.g., the breeding period or the last leg of spring migration). Furthermore, we highlight the complexity in daytime activities performed during the annual cycle, also showing how daytime activity is affected in different ways in relation to a high energy demanding activity such as flapping-flight migration. This information might help highlighting critical parts and bottlenecks of the annual cycle in migratory birds, when birds operate at increased levels of workload during specific energetically demanding periods.

## Supplementary Information

**Additional file 1 of** **Activity opatterns throughout the annual cycle in a long-distance migratory songbird, the red-backed shrike** ***Lanius collurio***. It contains the manuscript's supplementary information, including an example actogram, additional figures showing daytime activity during migratory periods and the activity values per hour throughout the annual cycle. Also, a summary table of daytime activity at each annual cycle period as well as information about the model outcome for daytime activity between males and females is provided.

## Data Availability

The datasets analysed for this study are available from the corresponding author upon request.
