# Supplementary material for: Activity patterns throughout the annual cycle in a long-distance migratory songbird, the red-backed shrike Lanius collurio
Source: Mov Ecol. 2022 Dec 1;10:55. doi: 10.1186/s40462-022-00355-0 (PMC9716747; doi:10.1186/s40462-022-00355-0)
Supplement: Supplementary file 1 — Additional file 1 of Activity opatterns throughout the annual cycle in a long-distance migratory songbird, the red-backed shrike Lanius collurio. It contains the manuscript's supplementary information, including an example actogram, additional figures showing daytime activity during migratory periods and the activity values per hour throughout the annual cycle. Also, a summary table of daytime activity at each annual cycle period as well as information about the model outcome for daytime activity between males and females is provided. [file 40462_2022_355_MOESM1_ESM.docx]

## Additional file 1 of “Activity patterns throughout the annual cycle in a long-distance migratory songbird, the red-backed shrike Lanius collurio”

Pablo Macias‑Torres, Thomas Alerstam, Arne Andersson, Johan Backman, Kasper Thorup, Anders P. Tottrup and Sissel Sjoberg

Figure S1. Migratory route of the red-backed shrikes and the actogram resulted from accelerometer data of a male monitored from 15 July 2019 until 7 July 2020. Orange line shows autumn migration while the blue line represents spring migration. On each side, half of the actogram is presented for one individual red-backed shrike recaptured in summer 2019. Each horizontal line in the actogram represents two consecutive days of data, divided by a black line (midnight), where the second day is repeated as the first day in the next line. Black arrows indicate the estimated geographical position of the main non-migratory periods of the studied population, inferred from the positions showed in Tøttrup et al., 2012. Autumn and spring migration are separated in three and two migratory periods respectively, deducted from the individual actograms. Each migratory period length is indicated by a small bracket of the same colour as the migratory period. In this particular actogram we observed two flights that were considered as odd flights as they did not fit into any migratory period (see methods).


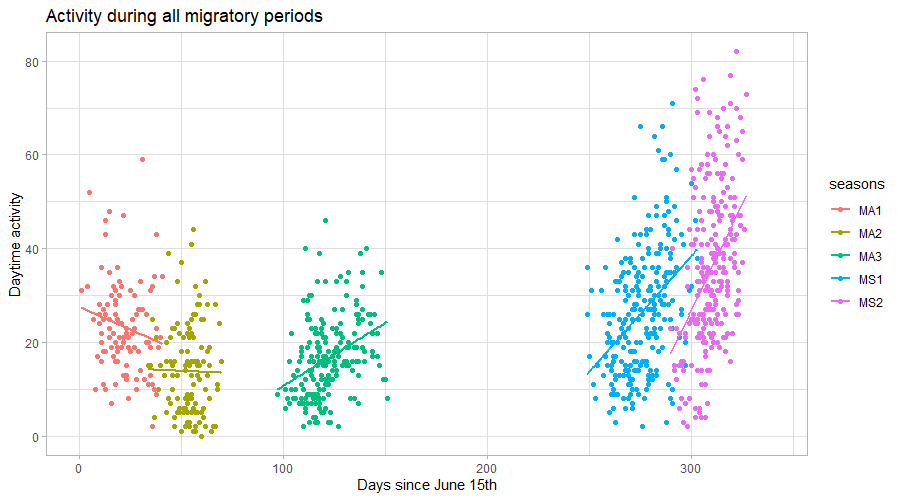


Figure S2. Daytime activity during migratory days for all the individuals. The regression line is the result of a linear model for each migratory segment.


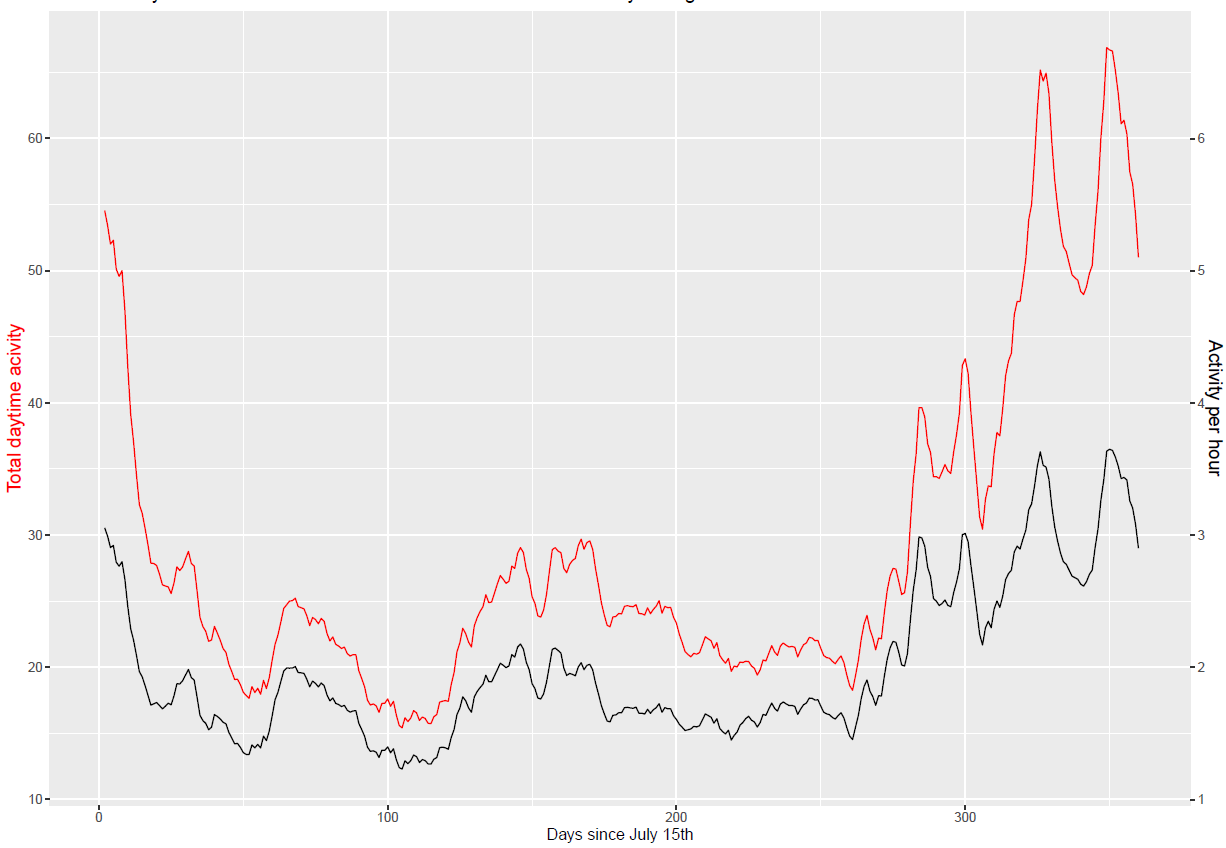


Figure S3. Total daytime activity and diurnal activity per hour in red and black respectively.

Table S1. Daytime activity values for each part of the annual cycle

| Periods | n | mean | SD |
| --- | --- | --- | --- |
| Individuals | 14 |  |  |
| MA1 | 109 | 23.3 | 9.85 |
| MA2 | 131 | 14.0 | 9.73 |
| MA3 | 201 | 16.6 | 8.68 |
| MS1 | 256 | 26.0 | 12.7 |
| MS2 | 245 | 35.5 | 16.3 |
| Stop and go spring | 251 | 21.6 | 8.86 |
| Stop and go fall | 178 | 32 | 14.6 |
| Stopover1 | 275 | 26.1 | 11.5 |
| Stopover2 | 680 | 21.2 | 8.9 |
| Stopover3 | 75 | 43.7 | 14.7 |
| Summer | 605 | 53.6 | 29.0 |
| Winter grounds | 1662 | 23.7 | 10.2 |
| #N/A (odd flights) | 40 | 23 | 12.8 |

Table S2. Pairwise differences for estimated marginal means between the four annual cycle events for females and males separately. This results from the model: sqrt(Activity) ~ events * Sex + daylength, random = ~1|Individual, correlation = corAR1(value = 0, form= ~day|Individual). The differences in marginal means are the result of subtracting the second parameter from the first one. Example: Autumn migration - Spring migration (in females) = -0.839 means that Spring migration marginal mean estimate is 0.839 larger than Autumn migration.

|  | estimate | SE | p value |
| --- | --- | --- | --- |
| Sex: Female | | | |
| Autumn migration - Spring migration | -0.839 | 0.178 | <.0001 |
| Autumn migration - Summer | 0.485 | 0.208 | 0.0922 |
| Autumn migration - Winter | 0.0552 | 0.147 | 0.982 |
| Spring migration - Summer | 1.324 | 0.227 | <.0001 |
| Spring migration - Winter | 0.894 | 0.177 | <.0001 |
| Summer - Winter | -0.430 | 0.209 | 0.168 |
| Sex: Male | | | |
| Autumn migration - Spring migration | - 0.714 | 0.102 | <.0001 |
| Autumn migration - Summer | -1.453 | 0. 141 | <.0001 |
| Autumn migration - Winter | -0.238 | 0.0791 | 0.0140 |
| Spring migration - Summer | -0.739 | 0. 148 | <.0001 |
| Spring migration - Winter | 0.476 | 0.101 | <.0001 |
| Summer - Winter | 1.215 | 0.137 | <.0001 |
